# Phlebotomine sand flies (Diptera: Psychodidae) and sand fly-borne pathogens in the Greater Mekong Subregion: a systematic review

**DOI:** 10.1186/s13071-022-05464-8

**Published:** 2022-10-05

**Authors:** John Hustedt, Didot Budi Prasetyo, Jodi M. Fiorenzano, Michael E. von Fricken, Jeffrey C. Hertz

**Affiliations:** 1 Vysnova Partners, AXA Tower, 8 Shenton Way, Level 34-01, Singapore, Singapore; 2 Entomology Division, Emerging Infections Department, U.S. Naval Medical Research Unit Two, Sembawang, Singapore, Singapore; 3 Department of Global and Community Health, College of Health and Human Services, George Mason University, Fairfax, VA USA

**Keywords:** Sand fly, Phlebotomine, *Leishmania*, Bartonellosis, Systematic review, Greater Mekong Subregion

## Abstract

Phlebotomine sand flies are proven or suspected vectors of several pathogens of importance, including leishmaniasis, bartonellosis and sand fly fevers. Although sand flies have a worldwide distribution, there has been limited research published on sand flies and sand fly-borne pathogens throughout the Greater Mekong Sub-region (GMS). This review followed the PRISMA guidelines to determine the biodiversity and presence of phlebotomine sand flies and their associated pathogens in the GMS, specifically Cambodia, Thailand, the Lao People’s Democratic Republic (Laos), Malaysia and Vietnam. A total of 1472 records were identified by searching electronic databases, scanning reference lists of articles and consulting experts in the field. After screening of title and abstracts, 178 records remained and were further screened for original data (*n* = 34), not having regional data (*n* = 14), duplication of data (*n* = 4), records not available (*n* = 4) and no language translation available (*n* = 2). A total of 120 studies were then included for full review, with 41 studies on sand fly-related disease in humans, 33 studies on sand fly-related disease in animals and 54 entomological studies focused on sand flies (5 papers contained data on > 1 category), with a majority of the overall data from Thailand. There were relatively few studies on each country, with the exception of Thailand, and the studies applied different methods to investigate sand flies and sand fly-borne diseases, impacting the ability to conduct meaningful meta-analysis. The findings suggest that leishmaniasis in humans and the presence of sand fly vectors have been reported across several GMS countries over the past 100 years, with local transmission in humans confirmed in Thailand and Vietnam. Additionally, local* Mundinia* species are likely transmitted by biting midges. Findings from this study provide a framework for future investigations to determine the geographic distribution and risk profiles of leishmaniasis and other associated sand fly-borne disease throughout the GMS. It is recommended that researchers expand surveillance efforts across the GMS, with an emphasis placed on entomological surveys, syndromic and asymptomatic monitoring in both humans and animals and molecular characterization of sand flies and sand fly-borne pathogens, particularly in the understudied countries of Cambodia, Vietnam and Laos.

**Graphical abstract:**

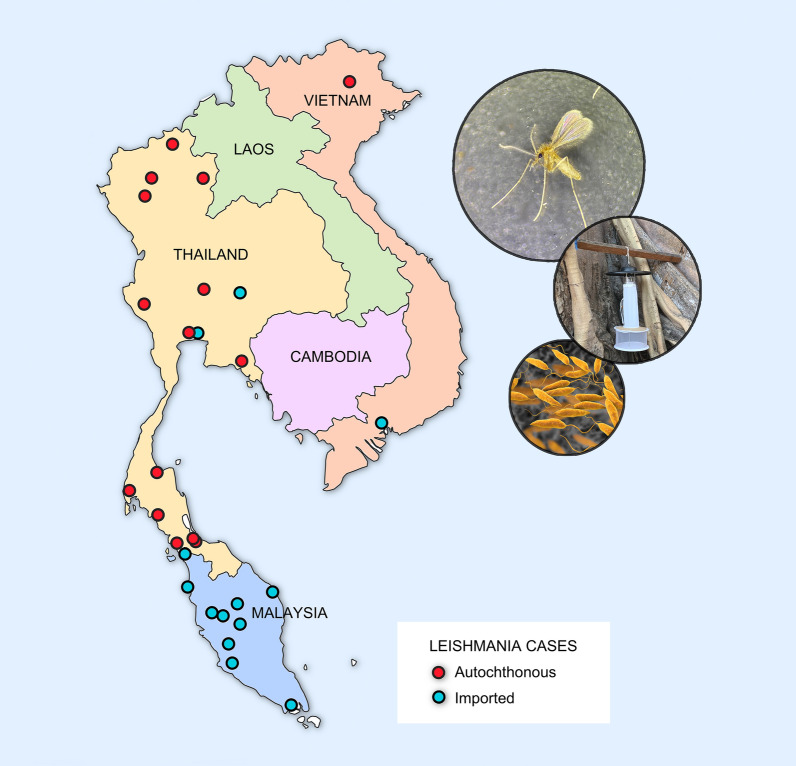

**Supplementary Information:**

The online version contains supplementary material available at 10.1186/s13071-022-05464-8.

## Background

Over the last century, there has been limited research published discussing sand flies (Diptera: Psychodidae, Phlebotominae) and sand fly-borne pathogens in the Greater Mekong Sub-region (GMS). Phlebotomine sand flies are medically-important biting insects, with more than 800 identified species, genera or subgenera, found in both temperate and tropical regions of the world [1]. They have a wide host range and are proven or suspected vectors of several pathogens of importance for both civilian and military personnel, including leishmaniasis, bartonellosis and sand fly fevers.

Leishmaniases are a group of diseases caused by the protozoan parasites belonging to the genus* Leishmania*. More than 20 *Leishmania* species are known to infect humans and are transmitted by the bite of infected female phlebotomine sand flies and some genera of biting midges [2–5]. There are three main types of leishmaniasis: (i) visceral leishmaniasis (VL), often known as “kala–azar” and the most serious form of the disease; (ii) cutaneous leishmaniasis (CL), the most common; and (iii) mucocutaneous leishmaniasis [4]. Leishmaniasis alone infects more than 12 million people globally, with 0.9–1.6 million new cases and 20–30,000 deaths each year [6]. In 2017, 94% of global VL cases were reported from seven countries: Brazil, Ethiopia, India, Kenya, Somalia, South Sudan and Sudan [7].

*Bartonella* is a genus of hemotropic, Gram-negative, aerobic bacilli bacteria with several species implicated in causing human and animal bartonellosis [8]. Infections can be widespread in domestic and wild animals, as the pathogens are capable of being transmitted by a variety of blood-feeding arthropods including sand flies, fleas, mites and ticks [8]. *Bartonella baciliformis*, also known as Oroyo Fever or Carrion’s disease in humans, is presumably transmitted by *Lutzomyia* spp. sand flies, particularly by *L. verrucarum* [9, 10], but limited information is available about the role of sand flies in transmission of other *Bartonella* species.

Diseases caused by sand fly-borne viruses remain neglected when compared to parasites such as leishmaniasis. Sand fly fevers, caused by *Phlebovirus* (Phenuiviridae), have a global distribution with four main serotypes: sand fly fever Sicilian virus (SFSV), sand fly fever Cyprus virus (SFCV), sand fly fever Naples virus (SFNV) and Toscana virus (TOSV) [11]. A more recently emerging virus from the Rhabdoviridae family, Chandipura virus (CHPV), has been recorded in the Indian subcontinent, Sri Lanka and Africa [12–15]. This virus causes encephalitis and is associated with a high fatality rate; there have been several outbreaks in the Indian subcontinent [16–19]. Like *Bartonella* infections, the overall disease burden and distribution of these sand fly-borne viruses remain poorly characterized in the GMS.

Historically, Southeast Asia has been considered to be leishmaniasis-free, with minimal emphasis placed on sand fly-borne disease and prevention. Recent detections in Thailand of autochthonous cases of a novel *Leishmania* pathogen in healthy individuals [20, 21], paired with detections of *Leishmania* DNA in vectors collected near human cases [22], have resulted in a renewed interest in sand fly research in the GMS. The purpose of this study was to compile and document what is known about sand flies and the diseases they carry in the GMS and provide recommendations for future research priorities in the region.

The objective of the review was: (i) to determine the biodiversity of phlebotomine sand flies and (ii) to determine the presence of the associated pathogens they carry in the GMS, specifically Cambodia, Thailand, the Lao People’s Democratic Republic (Laos), Malaysia and Vietnam.

## Methods

### Search strategy and eligibility criteria

This review follows Preferred Reporting Items for Systematic Reviews and Meta-Analyses (PRISMA) guidelines [23] and was performed between 1 March 2019 and 30 September 2019. All data were extracted by two independent researchers, with discrepancies resolved by consensus. The inclusion criterion was any study that reported primary data on the collection of sand flies and the presence of sand fly-borne illness (e.g. inclusive of detections in vector, humans and animals) in the GMS. Grey literature and unpublished data were included when identified, and no restrictions were set based on the language of the report or dates published. The exclusion criteria included any reviews or studies which report secondary data or did not contain primary data on sand fly collections or detections of sand fly-borne diseases.

### Data sources

Studies were identified by searching electronic databases, scanning reference lists of articles and through consulting with experts in the field. No limits were applied for language, with translation services and software applied to studies published in languages other than English. The search was conducted in PubMed, EMBASE, Web of Science, LILACS, Global Health and the Cochrane Database of Systematic Reviews. The term Lao was added to make sure all references to Laos or Lao PDR were captured. The following search terms were applied to all databases: “Phlebotomine sand*” AND (Cambodia OR Thailand OR Lao OR Malaysia OR Vietnam OR Mekong); “Sand fl*” AND (Cambodia OR Thailand OR Lao OR Malaysia OR Vietnam OR Mekong); Sandfl* AND (Cambodia OR Thailand OR Lao OR Malaysia OR Vietnam OR Mekong); Leishmania AND (Cambodia OR Thailand OR Lao OR Malaysia OR Vietnam OR Mekong); bartonellosis AND (Cambodia OR Thailand OR Lao OR Malaysia OR Vietnam OR Mekong); “sand* fevers” AND (Cambodia OR Thailand OR Lao OR Malaysia OR Vietnam OR Mekong); “Rift Valley Fever” AND (Cambodia OR Thailand OR Lao OR Malaysia OR Vietnam OR Mekong); Phlebovirus AND (Cambodia OR Thailand OR Lao OR Malaysia OR Vietnam OR Mekong); Sergentomyia AND (Cambodia OR Thailand OR Lao OR Malaysia OR Vietnam OR Mekong); Phlebotomus AND (Cambodia OR Thailand OR Lao OR Malaysia OR Vietnam OR Mekong).

### Study selection

For each search, titles and abstracts were imported into Mendeley reference manager software (Elsevier, Mendeley Ltd., Amsterdam, The Netherlands), duplicates were removed and titles and abstracts were screened. Full texts of potentially relevant records were retrieved and assessed for eligibility, utilizing interlibrary loan and attempting to contact authors directly when necessary to increase the total number of reviewed full text. Reference lists of all potentially eligible articles and reviews were also searched (Additional file 1: Table S1).

We developed data extraction sheets, pilot tested it on randomly selected included studies and refined accordingly (Additional file 2: Table S2). Data extracted for studies with entomology collections included: (i) species name of sand fly collected; (ii) density of sand flies collected; (iii) location collected; (iv) number of days of collection; (v) date collected; (vi) method of collection; (vii) sample size of sites; and (viii) any sand fly-borne pathogens detected. Data extracted for pathogen studies included: (i) type of sand fly pathogen detected; (ii) study design; (iii) target of study (e.g. sand fly/human/both); (iv) sample size; (v) location; (vi) date; and (vii) method used for detection of pathogens (Additional file 3: Table S3, Additional file 4: Table S4, Additional file 5: Table S5). One review author extracted data and a second author checked the extracted data. The primary outcomes were: (i) reported endemic species of sand flies; and (ii) the presence of sand fly-borne pathogens in the GMS.

The approach to synthesis included aggregating data at the study level and producing a quantitative and qualitative synthesis, with tables reporting all extracted data made using Excel (Microsoft Corp., Redmond, WA, USA). The qualitative synthesis attempted to aggregate information about each country, species of sand flies identified and detected pathogens. No statistical tests or meta-analysis were performed, with findings from this study providing primarily descriptive data to identify gaps in the literature. Disagreements were resolved by discussion between the two review authors; a third author resolved disagreements between the two review authors.

### Risk of bias

The commonly used guidelines for risk of bias in scientific studies (such as Effective Practice and Organization of Care [24] and Grading of Recommendations Assessment, Development and Evaluation [GRADE] [25]) are often focused on clinical trials in humans, and are not as useful when trying to determine bias in entomological laboratory studies. Therefore, any cluster randomized trial studies utilized the Cochrane risk of bias, but for all other studies we used a modified version of the GRADE guidelines [26] which rank a study as either high quality (further research is very unlikely to change our confidence in the estimate) to moderate quality (further research is likely to have an important impact on our confidence in the estimate and may change the estimate), low quality (further research is very likely to have an important impact on our confidence in the estimate and is likely to change the estimate) and very low quality (we are very uncertain about the estimate).

## Results

### Search results

**Fig. 1:**
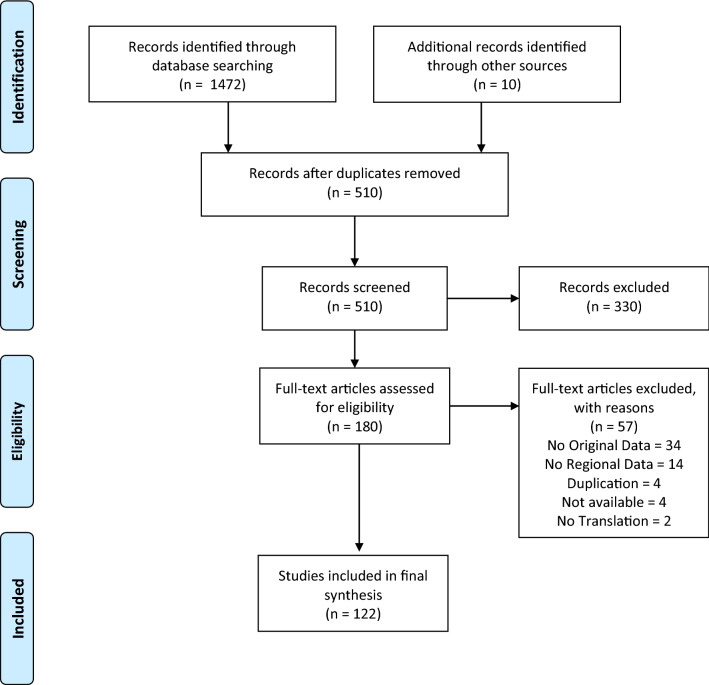
PRISMA diagram for a systematic review on Phlebotomine sand flies (Diptera: Psychodidae) and sand fly-borne pathogens in the Greater Mekong Sub-region

Search results, including inclusion and exclusion numbers, are illustrated in Fig. 1. Initially, 1472 records were identified through database searches, with 10 additional records identified through items that were shared internally and following the checking of references of included studies. After the screening of titles and abstracts, the remaining 178 records were assessed and reviewed in full, following which 58 articles were excluded. The reasons for exclusion were: (i) not having original data (*n* = 34); (ii) not having regional data (*n* = 14); (iii) duplication of data (*n* = 4); (iv) records not available (*n* = 4); and (v) no language translation available (*n* = 2). A total of 120 studies were then included in the review.

### Study characteristics

**Fig. 2:**
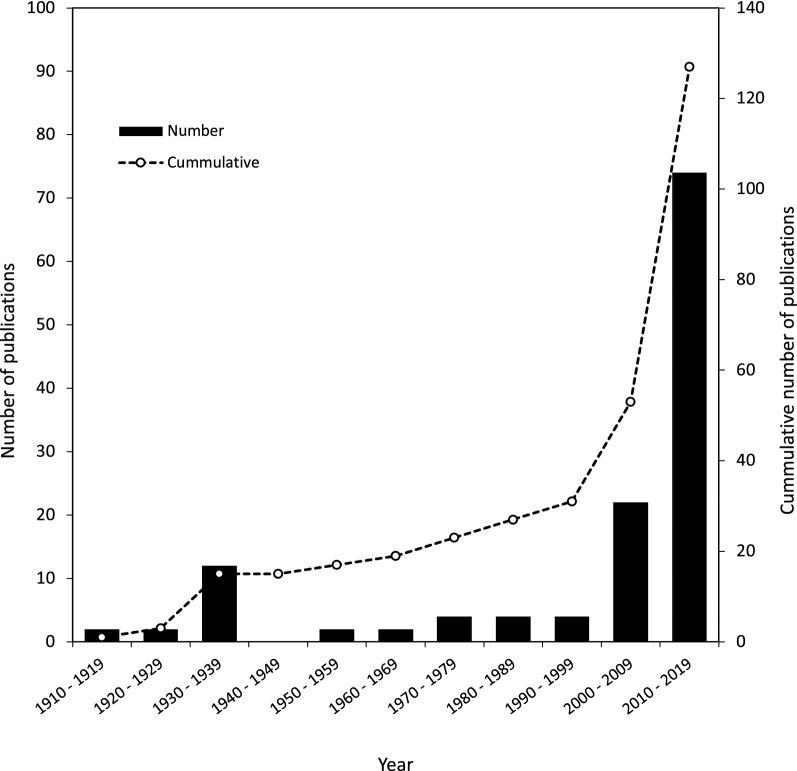
The number of sand fly and sand fly-borne disease articles published by year

**Fig. 3:**
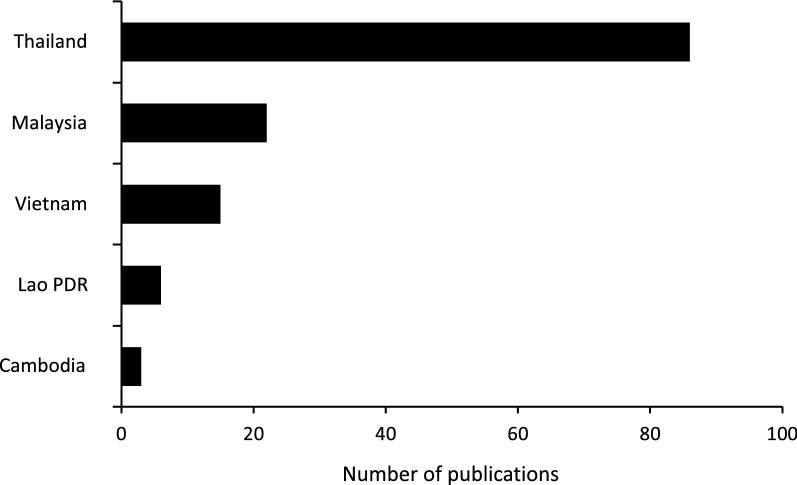
The Number of sand fly and sand fly-borne disease articles published by country

Included studies were published between 1914 and 2019 (Fig. 2), with most studies (58%) being published since 2010. Twelve studies were written in French, one in Thai, one in Norwegian and the remaining 106 in English. The majority of studies focused on Thailand (65%), followed by Malaysia (17%), Vietnam (11%), Laos (5%) and Cambodia (2%) (Fig. 3). All included studies were related to one or more of the following three core topics (5 studies included more > 1 core topic):

- Sand fly-borne disease in humans (41 studies) [20–22, 27–64]
- Sand fly-borne disease in animals (33 studies) [22, 38, 47, 65–93]
- Entomology studies on sand flies (54 studies) [22, 37, 38, 56, 94–143]

### Sand fly-borne disease in humans

References with human data were primarily case studies (*n* = 23) and cross-sectional surveys (*n* = 12), with the remaining seven studies being a mix of laboratory, retrospective and prospective studies (Additional file 1: Table S1). There were seven species of *Bartonella* and nine species of *Leishmania* reported (Table 1). The majority of included references were from Thailand (74%), with no pathogen studies reported in Cambodia or Vietnam. Older references reported unspecified sand fly-borne disease (e.g. “kala-azar,” Phlebotomous fever) but did not identify the actual pathogen causing the disease.

**Table 1:** Sand fly-borne disease pathogens reported in humans by country

Species	Distribution [with references]				
	Cambodia	Laos	Malaysia	Thailand	Vietnam
*Leishmania donovani*	–	–	–	[29, 45, 53, 54]	–
*L. infantum*	–	–	–	[35]	–
*L. lainsoni*^a^	–	–	–	[54]	–
*L. major*	–	–	–	[54]	–
*L. martiniquensis*	–	–	–	[21, 29, 37, 54, 57, 59]	–
*L. orientalis*	–	–	–	[55, 58, 63]	–
*L. siamensis*^b^	–	–	–	[20, 29, 52, 54, 60]	–
*L. tropica*	–	–	–	[53]	–
*Leishmania* spp.	–	–	[44, 50, 56]	[33, 42, 43]	–
CL (species identification not available)	–	–	–	[27]	–
VL (species identification not available)	–	–	–	–	[51]
Phlebotomus fever	–	–	[48]	–	–

*CL* Cutaneous leishmaniasis, *VL* Visceral leishmaniasis

^a^This species is endemic to the Neotropical region and the possibility that the parasite reported in Thailand is a different species cannot be ruled out

^b^Recently considered as invalid. This species might actually belong to *L. martiniquensis* and recently described *L. orientalis*[55]

**Fig. 4:**
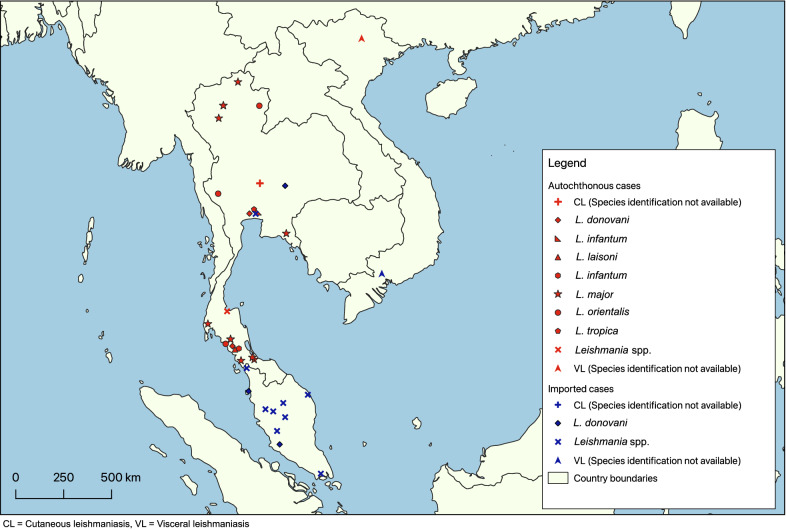
Map of sand fly-borne pathogens in humans

The first reports of leishmaniasis from Thailand were published by European physicians sent to combat plague in 1918 who noted the presence of both *L. donovani* and *L. tropica,* with the latter being found in more than 2% of patient ulcerations [53]. Despite this, recent papers in Thailand consider leishmaniasis to be an imported disease before 1999 [52]. From 1999 onwards, 22 laboratory-confirmed cases were reported (19 of which have been identified with molecular methods) [58], with *Leishmania martiniquensis* being the most common (15/22 cases) and the others associated with *Leishmania infantum* and *L. orientalis* [58]. The species *Leishmania siamensis* reported in earlier publications have been re-identified as *L. martiniquensis* using more comprehensive molecular methods, and since *L. siamensis* has never been formally described, the name is invalid and should not be used [21, 58]. *Leishmania martiniquensis* and *L. orientalis* were grouped together in *Mundinia*, a new subgenus recently introduced in 2016 [144, 145]. Members of this subgenus are presumably transmitted not only by sand flies but also by biting midges [2, 146]. Autochthonous symptomatic CL and VL cases have been reported sporadically in five southern, four northern, two central and one eastern province of Thailand [52, 55], and several additional asymptomatic cases among immunocompromised patients with human immunodeficiency virus/acquired immunodeficiency syndrome (HIV/AIDS) have occurred in nine provinces in southern Thailand [54] (Fig. 4).

Evidence for leishmaniasis transmission in other included GMS countries is lacking, with the majority of remaining references focusing on Malaysian immigrants [39, 44, 48, 50, 56]. A large cross-sectional study found high levels of *Leishmania* antibodies detected by RIDASCREEN® antibody enzyme-linked immunosorbent assay (ELISA) test kit in migrant workers from Myanmar (44%), Vietnam (26%) and Indonesia (26%) [56]. Two case reports of “kala–azar” were reported from Vietnam in 1931 and 1950 [49, 51]. However, no other studies on leishmaniasis were found from Vietnam, Laos or Myanmar throughout this review.

As previously mentioned, only *B. bacilliformis* has been linked to human transmission by sand flies and, as expected, there have been no reports of this bacterium in the GMS. However, other bartonelloses have been reported in the GMS, and many questions still remain regarding the maintenance and transmission of these pathogens. In 2000, *Bartonella henselae* was reported in 6.7% of healthy Thai blood donors, demonstrating the likelihood of frequent exposure across ages ranging from 20 to 70 years and in both sexes [41]. Subsequently, three novel *Bartonella tamiae* species were identified in 2002–2003 within a large prospective fever surveillance study in Chaing Rai and Khon Kaen, Thailand [34]. Findings indicated 7.7% positivity for *Bartonella* (*B. elizabethae, B. rattimassiliensi* and *B. tribocorum*, *B. henselae*, *B. vinsonii* and *B. tamiae*) by PCR among one study [31] and 12% positivity (*B. henselae*, *B. quintana*, *B. elizabethae*, *B. vinsonii* subsp. *vinsonii*) by immunofluorescence assay (IFA) in another study [28]. These results suggested that *Bartonella* is more common in rural Thailand than previously thought, although further evidence from an undifferentiated febrile illness study completed in 2017 in Nan Province only found 1% positivity by IFA. Additionally, a retrospective and subsequent prospective study in Laos, along the Thai border, found 7% positivity by IFA in patients admitted with definite or possible endocarditis [61]. These results, along with additional reports of *Bartonella* infections over various time points [30, 36, 62], suggest ongoing domestic transmission in humans within Thailand and likely Laos. Evidence for *Bartonella* transmission in the other GMS countries included in this review is limited, with only one case report of *B. henselae* and another case report of an unspecific *Bartonella* species reported from Malaysia [32, 40].

### Sand fly-borne disease reported in animals

Animal studies have only recently been reported, starting in 2001, with the majority of articles included in this review focusing on human or entomological investigations dating back almost 100 years. Animal studies in GMS are sparse and focused on *Leishmania,* predominantly reported from Thailand. In Thailand, *L. martiniquensis* was isolated in rodents [22], *L. dovovani* complex was isolated from both dogs and cats [73, 92] and *Leishmania* spp. was isolated in dogs, cats and buffalo [92]. *Leishmania donovani* complex (*L. infantum*) was isolated in a singular dog in Vietnam [147] (Table 2).

**Table 2:** Sand fly-borne disease pathogens reported in animals by country

Pathogens	Distribution [with references]				
	Cambodia	Laos	Malaysia	Thailand	Vietnam
*Leishmania siamensis*^a^	–	–	–	Rodents [22]	–
*L. donovani* complex	–	–	–	Dogs [92], cats [73]	–
*L. infantum*	–	–	–	–	Dogs [147]
*Leishmania* spp.	–	–	–	Dogs, cats, buffalo [92]	–

### Entomology studies on sand flies

Entomology studies on sand flies were largely broken up between identification keys (*n* = 26) and cross-sectional studies (*n* = 31). There were also two studies focused on laboratory methods from Thailand [98, 119] and two reports with unpublished primary data from Vietnam [104] and Laos [106]. The database of publications aggregated in this study indicate that most entomological activities regarding sand flies are focused on diversity rather than population control or insecticide resistance. However, one study in Thailand [111] did successfully establish a colony of wild-caught *Phlebotomus stantoni* and *Phlebotomus major major,* permitting further research on bionomic, vector competence and insecticide susceptibility status*.*

The sand flies found in the GMS are conservatively represented by three Old World genera: *Phlebotomus, Sergentomyia*, and *Chinius* [1]. Excluding taxonomic synonyms, 17 *Phlebotomus* species, including one unidentifiable species, 40 *Sergentomyia* species, including one unidentifiable species, and three *Chinius* species have been reported from the region (Table 3). Several studies documenting sand fly species diversity suggest that *Sergentomyia gemmea* is predominant in southern Thailand [103, 130]. However, recent molecular evidence raises concerns of misclassification for some of these *S. gemmea* specimens [136].

**Table 3:** Sand flies identified by country

Species	Distribution [with references]				
	Cambodia	Laos	Malaysia	Thailand	Vietnam
Phlebotomus					
* P. argentipes*	–	[138]	[123, 138]	[108, 130]	[138]
* P. asperulus*	–	–	[138]	[106, 108]	–
* P. barguesae*	–	–	–	[106, 135]	–
* P. betisi*	–	–	[138, 143]	[106]	[139]
* P. frondifer*	–	–	[138]	–	–
* P. hoeppli*	–	–	–	[106]	–
* P. kiangsuensis*	[100]	–	[138]	–	–
* P. longiforceps*	–	–	–	[141]	[139]
* P. major major*	–	–	[123]	[106–108, 124]	–
* P. mascomai*	–	–	–	[101, 106]	[139]
* P. nicolegerae*	[100]	-	–	–	–
* P. philippinensis gouldi*	–	–	–	[106, 108, 138]	–
* P. pholetor*	–	–	[138]	[106]	–
* P. stantoni*	[100]	[138]	[94, 138]	[106, 108, 138, 142]	[138, 139]
* P. teshi*	–	–	–	[106, 108, 124]	–
* P. yunshengensis*	–	–	–	–	[139]
* Larroussius* sp.	–	–	–	–	[139]
* Phlebotomus* sp. RP	–	–	–	[113]	–
Sergentomyia					
* S. anodontis*^a^	[100]	–	[138]	[108]	–
* S. bailyi*^a^	[100]	[138]	–	[138]	[138, 139]
* S. bandjara*	–	–	[138]	–	–
* S. barraudi*^a^	[138]	[138]	–	[107, 108, 138]	[138, 139]
* S. barraudi* RP	–	–	–	[113]	–
* S. brevicaulis*	–	–	–	[106]	[138, 139]
* S. cheongi*	–	–	[94]	–	–
* S. dentata*	–	–	–	[106–108]	–
* S. gemmea*	–	[136]	[138]	[126, 136]	–
* S. gombaki*	–	–	[138]	[134]	–
* S. hamidi*	–	–	[138]	–	–
* S. hivernus*^a^	–	[136]	–	[136, 138]	[138, 139]
* S. hodgsoni*	–	–	[123]	[106, 108]	–
* S. indica*^a^	[138]	–	[123, 138]	[106, 108, 130, 138]	–
* S. iyengari*^a^	–	[136, 138]	[138]	[106, 108, 130, 138]	[138, 139]
* S. jefferyi*	–	–	[138]	–	–
* S. kelantani*	–	–	[138]	–	–
* S. khawi*	[100]	[136, 138]	–	[105, 136]	–
* S. knudseni*	–	–	[94, 138]	–	–
* S. malayae*	–	–	[138]	–	–
* S. mahadevani*	–	–	–	[108, 138]	–
* S. morini*^a^	–	–	–	–	[138]
* S. pachystoma*	–	–	[138]	–	-
* S. perturbans*^a^	[138]	[138]	[138]	[106, 108, 130]	[138, 139]
* S. phadangensis*	–	–	–	[112]	–
* S. phasukae*	–	–	–	[134]	–
* S. punjabensis*	–	–	–	[106, 108]	–
* S. quatei*	–	–	[123, 138]	[106, 108]	–
* S. raynali*	–	[136]	–	[136]	–
* S. reidi*	–	–	[123]	–	–
* S. rudnicki*	–	–	[138]	–	–
* S. silvatica*^a^	[138]	[138]	[123, 138]	[106, 130, 138]	[138, 139]
* S. tambori*	–	–	[123, 138]	–	–
* S. tonkinensis*	–	–	–	–	[138]
* S. traubi*	–	–	[138]	–	–
* S. whartoni*	–	–	[138]	–	–
* S. sepilok*	–	–	[123, 138]	–	–
* S. linearis*	–	–	[123]	–	–
* Sergentomyia* sp. RP	–	–	–	[113]	–
Chinius					
* C. barbazani*	–	–	–	[106, 108, 137]	–
* C. eunicegalatiae*	–	[95]	–	–	–
* C. junlianensis*	–	–	–	–	[139]

^a^Previously listed as *Phlebotomus* in older publications

Species richness per country largely paralleled the number of entomology studies conducted (Thailand > Malaysia > Vietnam > Laos > Cambodia). Thailand and Malaysia recorded 34 and 32 species, respectively, compared to the number of species reported from Vietnam (*n* = 18), Cambodia (*n* = 10) and Laos (*n* = 8). *Phlebotomus stantoni* was the only species recorded in all countries, but presumably this is an artifact of the limited surveillance conducted in the region. For example, *Sergentomyia barraudi* was collected in every country but Malaysia. Similarly, *Sergentomyia iyengari* was reported everywhere except Cambodia; Laos was the only country yet to report *Sergentomyia perturbans* and *Sergentomyia silvatica*.

At the time of writing this report, Malaysia and Thailand were the only countries in the region that have reported *Leishmania* screening in their sand fly populations. *Leishmania martiniquensis* DNA has been detected in three *Sergentomyia* species from Thailand: *S. barraudi*, *S. gemmea* and *S. iyengari* [122]. Among these species, human DNA was been detected in the blood meal of *S. gemmea* and *S. iyengari,* suggesting that these species also feed upon humans. However, recent molecular evidence raises concerns of misclassification for some of these *S. gemmea* specimens [136]. In Malaysia, none of the sand flies collected in one study from 10 districts in Peninsular Malaysia tested positive for *Leishmania* DNA [94]. While the presence of both phlebotomine sand flies and the sand fly-borne pathogens have been reported in some Mekong countries, information remains sparse due to the absence of strong surveillance systems and limited laboratory resources for analysis.

## Discussion

An increasing number of autochthonous VL cases have been reported in Thailand since 1999 [58], which in turn may have led to a disproportionate number of studies occurring in Thailand. The results of this review also suggest that bartonellosis does not appear to be a medically important sand fly-associated disease in the GMS region. However, the lack of knowledge on the hosts, vectors and transmission pathways of other *Bartonella* spp. in this region warrants further investigation.

The first reports of endemic human leishmaniasis in GMS occurring in Thailand came in 1918 [53], with additional reports in Vietnam as early as 1931 [51]. Despite these reports, leishmaniasis was considered to be an imported disease in Thailand until 1999 [52, 58]. Leishmaniasis in non-human animals was first reported in 2001, with most studies focused on rodents, dogs, cats and buffalos in Thailand. Older references reported “Kala Azar” or “Phlebotomous fever” but they did not specifically identify the pathogen. These findings suggest that leishmaniasis has been detected in several GMS countries over the past 100 years, with local transmission confirmed in Thailand and Vietnam. These findings pose a larger question about the burden and risk of leishmaniasis in the GMS and whether it is possibly re-emerging over the past decade, or if it has remained endemic and has simply been neglected from a research and surveillance perspective?

Entomology studies throughout Southeast Asia have detected leishmaniasis in both humans and animals in Southeast Asia, with the sand fly vectors of the disease identified regionally over the past 100 years. A concerted effort is needed to determine species richness, syndromic surveillance and serological monitoring in GMS countries other than Thailand, to fill in major gaps regarding sand fly-borne diseases in Southeast Asia [148, 149]. The absence of data regarding sand fly-borne diseases in humans may also limit targeted vector surveillance efforts; for example, a lack of leishmaniasis cases recorded in humans may result in entomology studies not being prioritized over more pressing disease vectors such as *Anopheles* and *Aedes* mosquitoes. There may be unreported sand fly species capable of vectoring *Leishmania* and *Bartonella* pathogens throughout the region that warrant updated investigations that utilize various modern molecular methods for species identification and pathogen detection. However, caution should be taken when using the detection of the pathogen’s DNA to suggest vectors are transmitting the pathogens to animals as the presence of pathogen DNA in an arthropod’s body does not provide evidence on the viability of the parasite or its ability to infect the host [146].

An important aspect of sand fly biology is their anthropophilic behavior. Most of the entomological studies reviewed were carried out using CDC light traps as a sole collection device. Studies in Thailand and Malaysia have included human landing collection as an additional collection method to better understand host preference of sand flies [38, 96, 106, 123]; similar studies are needed in Laos, Cambodia and Vietnam. A more recent effort by Siripattanapipong et al. [126] determined sand flies’ blood meal preferences using DNA sequencing technology, with the authors reporting the detection of human DNA in *P. stantoni*, *S. barraudi*, and *S. iyengari* blood meals. Further research is needed to see if host availability or certain abiotic factors affect blood meal preference in sand flies and how this impacts disease transmission risk in GMS.

Zoonotic disease transmissions can be exacerbated by large populations of freely roaming animals, increased pet ownership (e.g. cats, dogs) and the presence of agricultural animals living in close proximity to humans, as seen in many regions in Southeast Asia [147]. Throughout this sand fly review, there were very few manuscripts that addressed sand fly pathogens in animals, with most research coming from Thailand and focusing on leishmaniasis. Sand fly-associated pathogens, such as Phleboviruses (Salehabad virus and sand fly fevers) and CHPV occur throughout Asia and are addressed in many research papers examined during this review; however the roles animals play as reservoirs is not clear [150]. Research into sand fly-associated pathogens in animals throughout GMS could lead to an understanding of the transmission cycles and enable better integrated vector management techniques.

In the window of time between conducting this systematic review and publishing this manuscript, eight additional papers have been published related to sand flies and leishmaniasis in the GMS. One study on the prevalence and associated risk factors of *Leishmania* infection among immunocompetent hosts in Thailand focused on an outbreak investigation of an index case of CL caused by *L. martiniquensis* in 2015 and found that 7.1% of 392 participants were positive for *Leishmania* infection [151]. One participant tested positive with *L. martiniquensis,* while the rest were positive with either *L. orientalis* or *Leishmania* spp*.* The most statistically significant risk factor was having domestic animals in the housing area, indicating a sevenfold greater risk of infection. At the index patient’s school, seven *S. gemma and* one *P. stantoni* sand fly samples and one black rat were positive for *Leishmania* spp. However, the occurrence of these species in humans and animals, as well as in the sand fly species identified, have all been previously reported in other studies in Thailand. The additional seven papers included one report on the detection of *Bartonella* in Vietnamese rodents [152], one on Vietnamese sand fly collections [153] and five on Thai sand fly collections [154–158]. In these seven papers, three new species of sand fly species were reported, including *Sergentomyia rudnicki* [154] (2 samples) and *Phlebotomus papatasi* [157] (16 samples) in Thailand, and *Sergentomyia khawi* [153] (27 samples) in Vietnam. While not substantially changing the findings of this body of work, these additional studies reiterate the need to expand sand fly entomological surveillance and conduct research targeting leishmaniasis to areas outside of Thailand in the GMS region.

There were several limitations to this study, many of which are common in systematic reviews. Each country, with the exception of Thailand, had relatively few studies and applied different methods to investigate sand flies and sand fly-borne diseases, impacting our ability to conduct a meaningful meta-analysis. Another limitation was that many of the studies occurred several decades ago, when molecular methods had not been developed, leading to potential risks of vector misclassification and underestimation of disease burden. Furthermore, land use and deforestation in the GMS has drastically changed the environments where many of these vector studies originally occurred over 50 years ago, warranting a re-examination of sand fly-borne diseases and vector ecology for the greater region.

## Conclusions

Findings from this study may help future investigations on the epidemiology of leishmaniases to determine the geographic distribution and risk profiles of leishmaniasis and other associated sand fly-borne disease throughout the GMS. Updated entomology keys are also needed within the GMS as the last regional sand fly key was published in the 1930s. It is recommended that researchers expand surveillance efforts across the GMS, with an emphasis placed on entomological surveys, syndromic and asymptomatic monitoring in both humans and animals and molecular characterization of sand flies and sand fly-borne pathogens, particularly in the understudied countries of Cambodia, Vietnam and Laos.

## Supplementary Information

**Additional file 1: Table S1.** Bartonellosis reported in humans.

**Additional file 2: Table S2.** Bartonellosis reported in animals.

**Additional file 3: Table S3.** Data extracted from the entomology papers included in this review.

**Additional file 4: Table S4.** Data extracted from the animal epidemiology papers included in this review.

**Additional file 5: Table S5.** Data extracted from the human epidemiology papers included in this review.

## Data Availability

All data generated or analyzed during this study are included in this published article and its Supplementary Information files.

## Abbreviations

CL: Cutaneous leishmaniasis
ELISA: Enzyme-linked immunosorbent assay
GMS: Greater Mekong Sub-region
HIV/AIDS: Human immunodeficiency virus/acquired immunodeficiency syndrome
IFA: Immunofluorescent assay
PRISMA: Preferred Reporting Items for Systematic Reviews and Meta-Analyses
VL: Visceral leishmaniasis
