# Correction: Phlebotomine sand flies (Diptera: Psychodidae) and sand fly-borne pathogens in the Greater Mekong Subregion: a systematic review

**DOI:** 10.1186/s13071-022-05550-x

**Published:** 2022-11-17

**Authors:** John Hustedt, Didot Budi Prasetyo, Jodi M. Fiorenzano, Michael E. von Fricken, Jefrey C. Hertz

**Affiliations:** 1 Vysnova Partners, AXA Tower, 8 Shenton Way, Level 34-01, Singapore, Singapore; 2 Entomology Division, Emerging Infections Department, U.S. Naval Medical Research Unit Two, Sembawang, Singapore, Singapore; 3 Department of Global and Community Health, College of Health and Human Services, George Mason University, Fairfax, VA USA

## Correction: Parasites & Vectors (2022) 15:355 10.1186/s13071-022-05464-8

Following publication of the original article [1], it came to the authors’ attention that they had not provided an up-to-date version of Figure 1, with the result that there was a mismatch between the figure and the ‘Search results’ section of the Results. The article has since been updated with the correct figure and the correct figure (Fig. 1) may be seen in this erratum.

**Fig. 1:**
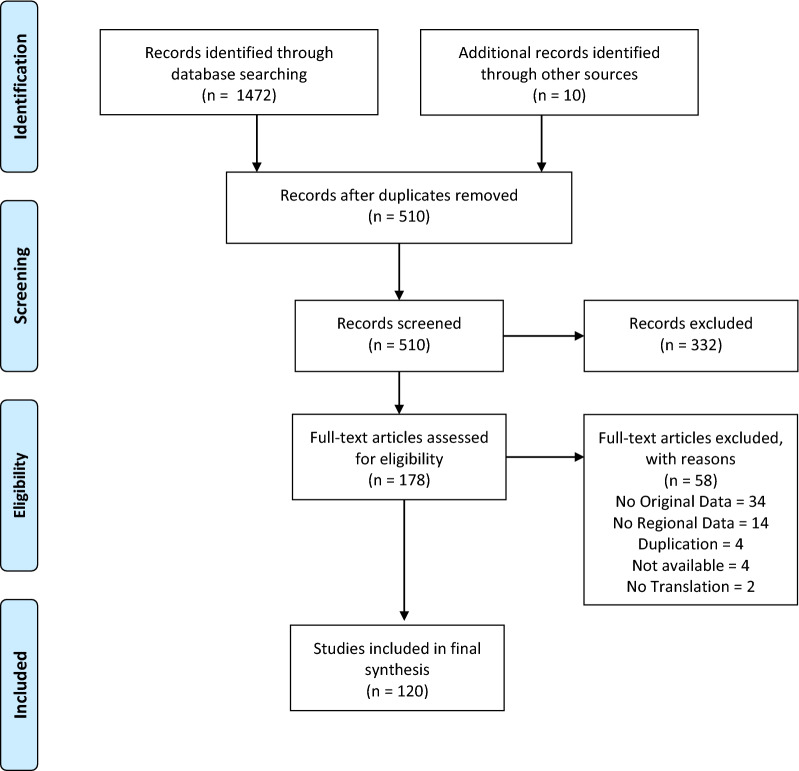
PRISMA diagram for a systematic review on Phlebotomine sand flies (Diptera: Psychodidae) and sand fly-borne pathogens in the Greater Mekong Sub-region

The authors thank you for reading and apologize for any inconvenience caused.
